# Supplementary material for: Prediction of individual mortality risk among patients with chronic obstructive pulmonary disease: a convenient, online, individualized, predictive mortality risk tool based on a retrospective cohort study
Source: PeerJ. 2022 Dec 6;10:e14457. doi: 10.7717/peerj.14457 (PMC9745921; doi:10.7717/peerj.14457)
Supplement: Supplemental Information 1 [file peerj-10-14457-s001.doc]

Gender:0 for female; 1 for male

Consciousness:0 for sober;1 for Lethargy; 2 for Blurred consciousness;3 for coma prophase; 4 for coma

Survival_status: o for survival; 1 for dead

Center: o for Shunde Hospital, Southern Medical University; 1 for The Affiliated Chencun Hospital of Shunde Hospital, Southern Medical University

Group: o for model group; 1 for validation group.
